# Effectiveness of Oxygen during Sintering of Silver Thin Films Derived by Nanoparticle Ink

**DOI:** 10.3390/nano12111908

**Published:** 2022-06-02

**Authors:** Feng Feng, Haofeng Hong, Xing Gao, Tian Ren, Yuan Ma, Pingfa Feng

**Affiliations:** 1 Division of Advanced Manufacturing, Shenzhen International Graduate School, Tsinghua University, Shenzhen 518055, China; feng.feng@sz.tsinghua.edu.cn (F.F.); hhf19@mails.tsinghua.edu.cn (H.H.); rt20@mails.tsinghua.edu.cn (T.R.); 2 Shenzhen Jinzhou Precision Technology Corp., Shenzhen 518116, China; 3 Shenzhen Tsingding Technology Co., Ltd., Shenzhen 518133, China; ma.yuan@tsingding.com; 4 Department of Mechanical Engineering, Tsinghua University, Beijing 100084, China

**Keywords:** silver thin film, nanoparticle (NP) ink, sintering, oxygen, polyvinylpyrrolidone (PVP), dispersant decomposition, electrical resistivity

## Abstract

Silver nanoparticle (NP) inks have been widely used in the ink-jet printing field because of their excellent properties during low-temperature sintering. However, the organic dispersant used to prevent the aggregation and sedimentation of NPs can hinder the sintering process and result in the high resistivity of sintered films. In this study, silver thin films derived from silver NP ink with polyvinylpyrrolidone (PVP) dispersant were sintered in different atmospheres of pure nitrogen, air, and pure oxygen. The effect of the oxygen content in the sintering atmosphere on the thermal properties of the ink, the electrical resistivity and microstructure of the sintered films, and the amount of organic residue were studied by using differential scanning calorimetry, the four-point probe method, scanning electron microscopy, Fourier transform infrared spectroscopy, etc. The mechanism of optimizing the film resistivity by influencing the decomposition of the PVP dispersant and the microstructure evolution of the silver thin films through the sintering atmosphere was discussed. The results demonstrated that an oxygen-containing atmosphere could be effective for silver NPs in two ways. First, the oxygen content could enhance the diffusion ability of silver atoms, thus accelerating the stage transition of microstructural evolution at low temperatures. Second, the oxygen content could enable the PVP to decompose at a temperature much lower than in conditions of pure nitrogen, thus helping to finalize the densification of a silver film with a low resistivity of 2.47 μΩ·cm, which is approximately 1.5-fold that of bulk silver. Our findings could serve as a foundation for the subsequent establishment of ink-jet printing equipment and the optimization of the sintering process for printing silver patterns on flexible substrates.

## 1. Introduction

The direct printing of conductive ink has emerged as an important alternative to traditional lithography, which is a complex multistep method for producing conductive thin films or patterns that generates a lot of waste [1]. Many examples of the use of metallic inks in printed electronics have been reported, such as printed circuit boards [2,3,4], light-emitting diodes (LEDs) [5,6], flexible displays [7,8], radio frequency identification (RFID) tags [9,10], solar cells [11,12], and transparent electrodes [13,14].

Regarding printing techniques, metal can be printed by using inks containing metallic nanoparticles (NPs) or low-viscosity inks with organometallic precursors; then, sintering is carried out to form the conductive thin films or patterns. NP-based inks are more commonly used because they can achieve a low electrical resistivity, i.e., a high conductivity, due to their higher solid content [15]. Such a technique is efficient in terms of material utilization, scalable and simple in the terms of processing, and adaptable to various types of substrates [16,17,18]. 

Many metals have available NP inks, such as silver [1,19], gold [20,21], and copper [22,23,24]. Silver-based inks are currently preferred due to their excellent electrical conductivity and stability in air, and silver is relatively inexpensive compared to gold [16,25]. Additives such as polyvinylpyrrolidone (PVP) [26,27,28], polyacrylic acid (PAA) [8,29,30], 2-amino-2-methyl-1-propanol (AMP) [31], and tetradecane [32] are generally added to improve the ink’s uniformity during preparation, avoiding aggregation and thus improving the reliability of the printing process. 

The sintering of printed metal NPs is necessary to obtain a high conductivity. The NP coalescence of a metal usually occurs at temperatures far below its bulk-form melting temperature, because there is a strong correlation between the size and the melting point of NPs, which can be derived by surface energy and thermodynamic theory [33]. During sintering, a higher conductivity can be achieved by improving the contact between NPs, which increases the removal of the organic additives [34], and so a higher sintering temperature is generally required [35]. However, for flexible substrates, the sintering temperatures cannot be too high, as this would damage the substrates, resulting in poor electrical conductivity due to the residual organic additives, particularly the dispersant with its high decomposition temperature.

Different methods have been proposed to solve the above problem, e.g., modifying the surface of silver NPs [36], using chemical reagents to remove the dispersant on the surface of silver NPs [37,38], thinning the dispersant layer on the NP surface [17], and using a dispersant with a low decomposition temperature [39,40]. These methods generally focus on the optimization of the organic dispersant, but no previous publication has reported a systematic study on the effect of an oxygen-containing atmosphere on sintered silver thin films. In this study, the effects of different sintering atmospheres on the microstructure and electrical properties of silver thin films were studied by using a silver NP ink with a PVP dispersant. It was found that the oxygen content in the sintering atmosphere could effectively promote the grain size enlargement and the decomposition of the PVP dispersant, resulting in a favorable electrical conductivity, which could provide a new basis for fabricating high-performance conductive thin films and developing practical equipment for ink-jet printing on flexible substrates.

## 2. Experimental Details

The ink used in this study was composed of silver NPs that were uniformly dispersed with the aid of a PVP dispersant in a solution containing isopropanol and diethylene glycol methyl ether. The viscosity of the ink measured by a viscometer (LAWSON, DHJ-5S, Ningbo, China) was 10 cp at 25 °C, and the surface tension of the ink measured by the automatic surface tension meter (Zibo Boshan Haifen Instrument Factory, HZ-800, Zibo, China) was 29 mN/m at 25 °C.

The thermodynamic properties of the ink during the thermal sintering process were characterized by using a differential scanning calorimeter (DSC, Mettler-Toledo, DSC3, Zurich, Switzerland), in which the ink was heated from room temperature to 600 °C at a ramp rate of 10 °C·min^−1^, and the mass change of the sample, i.e., the thermogravimetric (TG) data, and the DSC data were recorded.

**Figure 1:**
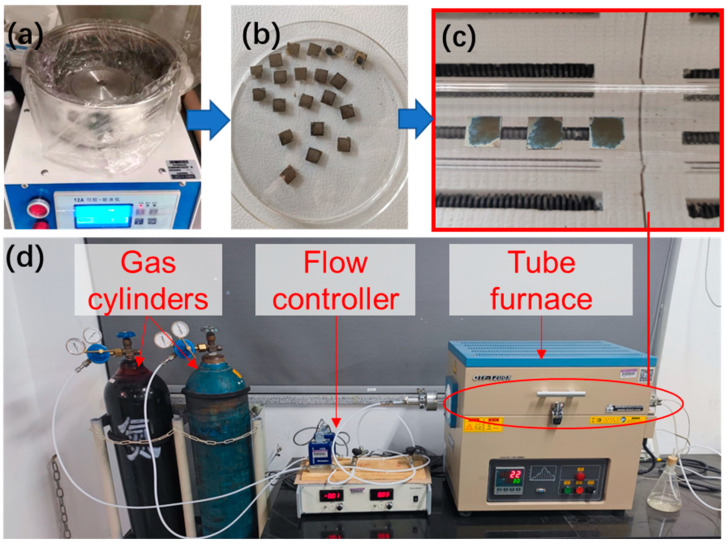
Flow chart of sample preparation and sintering. (**a**,**b**) Preparation of silver thin films to be sintered using a homogenizer; (**c**) thin films sintered in a tube furnace; (**d**) sintering equipments.

A flow chart of the sample preparation and sintering process is shown in Figure 1. The ink was spin-coated on a square glass sheet with a side length of 10 mm by a homogenizer (Zhangqiu Crown, 12A, Zhangqiu, China) and then dried in an oven at 75 °C for 10 min. The coated samples were heat-treated in a tube furnace (HF-Kejing, OFT-1200X, Hefei, China) under atmospheres of various oxygen contents and temperatures for 30 min. The oxygen content of the sintering atmosphere was controlled by the mass flow controller (Sevenstar, D07, Beijing, China), which uses two channels of oxygen (99.999% pure) and nitrogen (99.999% pure); the oxygen contents used in this study were 0%, 21% (denoted as air hereafter), and 100%. The heat treatment temperature ranged from 150 °C to 450 °C.

The electrical resistivity of the sintered samples was measured by a four-point probe meter (HELPASS, HPS2526, Changzhou, China). The microscopic morphology of the films was characterized using a scanning electron microscope (SEM, Carl Zeiss, ZEISS SUPRA 55, Oberkochen, Germany), and both the surface and cross-section of the films were observed. Fourier transform infrared (FTIR) spectroscopy (Nicolet, iS50, Waltham, MA, USA) was used to detect the residual organic matter in the samples after sintering. 

## 3. Results

### 3.1. Thermal Analysis of Silver NP Ink

**Figure 2:**
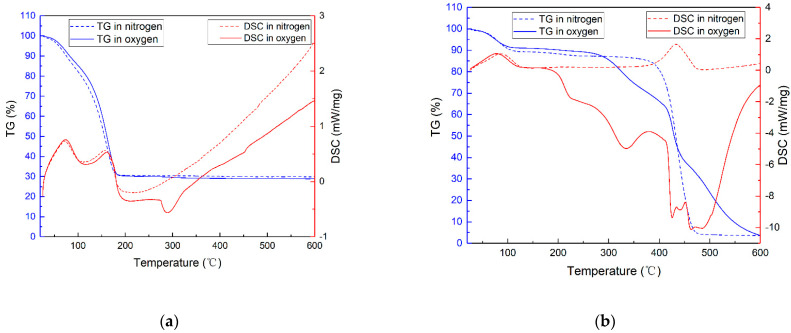
(**a**) TG−DSC curves of silver NP ink in the atmospheres of pure oxygen and pure nitrogen; (**b**) TG−DSC curves of silver PVP K30 in the atmospheres of pure oxygen and pure nitrogen.

The thermal analysis results for the ink are illustrated in Figure 2a. It could be observed that the sample mass decreased as the temperature gradually increased from room temperature, and a mass reduction of 69.17% at 184 °C was recorded under both atmospheres of pure oxygen and pure nitrogen. The mass loss measured in the TG analysis at this stage was caused by the volatilization of the solvents isopropanol and diethylene glycol methyl ether in the ink, which corresponded to the two endothermic peaks in the DSC results located at about 70 °C and 160 °C, respectively.

The sample mass under both atmospheres changed slowly above 200 °C. The TG data indicated that the total mass of the samples in pure oxygen decreased by 0.954% from 200 °C to 600 °C, while that in pure nitrogen only decreased by 0.485%. The DSC curves under the two atmospheres were different above 200 °C. There was an obvious exothermic peak between 280 °C and 330 °C in pure oxygen, which was caused by the separation and combustion of the organic dispersant coated on the silver NPs, based on the report by Yan et al. [41]. In order to verify that the exothermic peak originated from the oxidative decomposition of PVP, a thermal analysis of PVP K30 (*M*_W_ = 40,000), which is often used as an auxiliary for reducing silver nanowire and as a stabilizer for silver NPs in inks [42], was carried out and is shown in Figure 2b. It could be seen that the PVP in oxygen experienced an exothermic phenomenon from 200 °C, with several exothermic valleys around 300–500 °C, and the mass loss at this stage was significant. The endothermic phenomenon in this temperature range is related to the breaking and decomposition of PVP [43,44]. The mass loss in nitrogen was concentrated around 450 °C, and the DSC indicated that there was an endothermic phenomenon at this temperature. Therefore, it could be inferred that PVP endothermically decomposed above 400 °C in an oxygen-deficient environment, while the presence of oxygen caused the decomposition of PVP at temperatures around 300 °C.

As shown in Figure 2a, the mass loss of the ink in the endothermic peak stage around 300 °C was 0.60%, which constituted the majority of the mass loss above 200 °C. However, the DSC curve in pure nitrogen did not show similar changes. It was found that the endothermic and exothermic conditions and mass loss of the ink and PVP were consistent, and it could be determined that the mass loss above 200 °C in the silver nanoparticle ink was mainly due to the oxidative decomposition of the organic dispersant PVP. It is worth mentioning that there is a certain difference in the endothermic peak positions between the ink and PVP, which may be due to the difference in the molecular weight of PVP and the change in properties caused by the extremely thin PVP wrapped around the NPs.

The DSC and TG data implied that the solvents in the ink could volatilize completely through sintering at 200 °C, but the removal of the dispersant PVP was much more difficult. Even in the pure oxygen atmosphere, PVP could only be removed above 330 °C.

### 3.2. Enhancement of Film Conductivity

**Figure 3:**
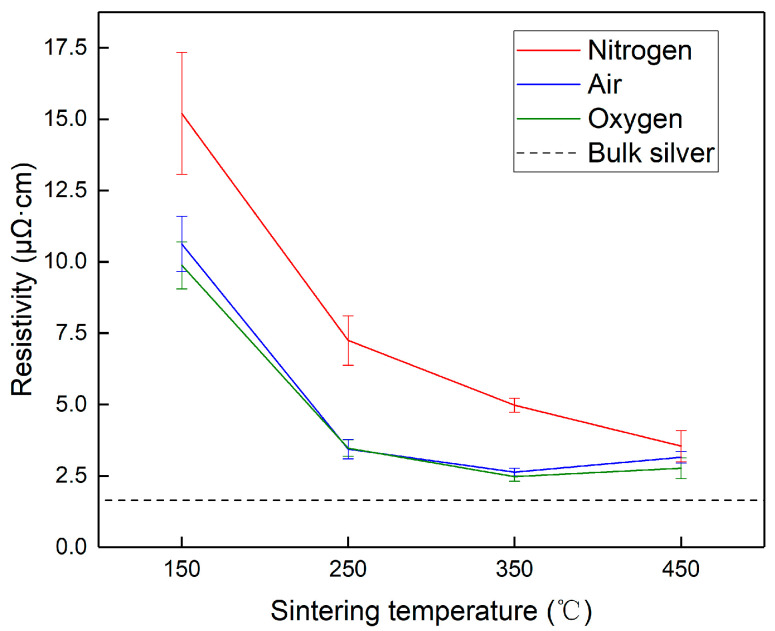
The resistivity curves under various sintering atmospheres and temperatures.

The resistivity of the films sintered under different atmospheres and temperatures is plotted in Figure 3. A significant reduction in resistivity could be observed as the sintering temperature increased from 150 °C to 350 °C under all the atmospheres. The lowest resistivity was 2.47 μΩ·cm, which was approximately 1.5-fold that of bulk silver. The resistivity curves in air and pure oxygen were quite similar, and there was a slight rise in resistivity at the sintering temperature of 450 °C. Under the atmospheres of pure nitrogen, the resistance continued to decrease at the sintering temperature of 450 °C. Generally, the resistivity of the films sintered under an atmosphere containing oxygen was much lower than those without oxygen. Therefore, it can be summarized that oxygen promoted the high electrical performance of the sintered films. Moreover, the small difference between the curves of pure oxygen and air might be due to the fact that the amount of oxygen supplied in both atmospheres was much higher than the reaction demand for the decomposition of PVP.

### 3.3. Microstructure of Sintered Films

**Figure 4:**
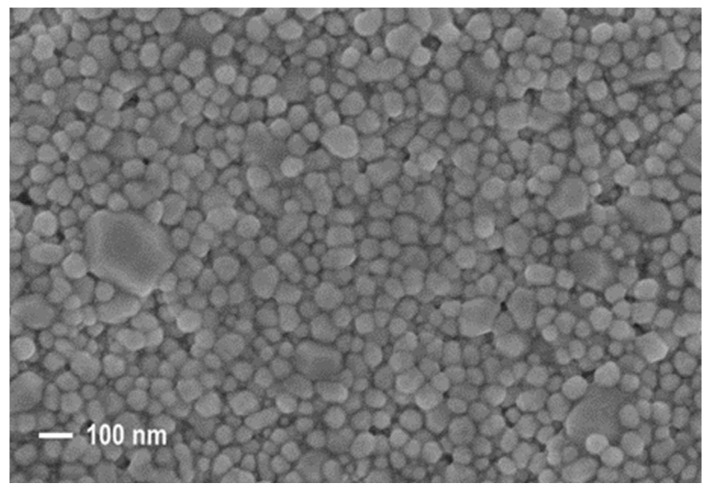
SEM image of silver NPs after drying at 75 °C.

**Figure 5:**
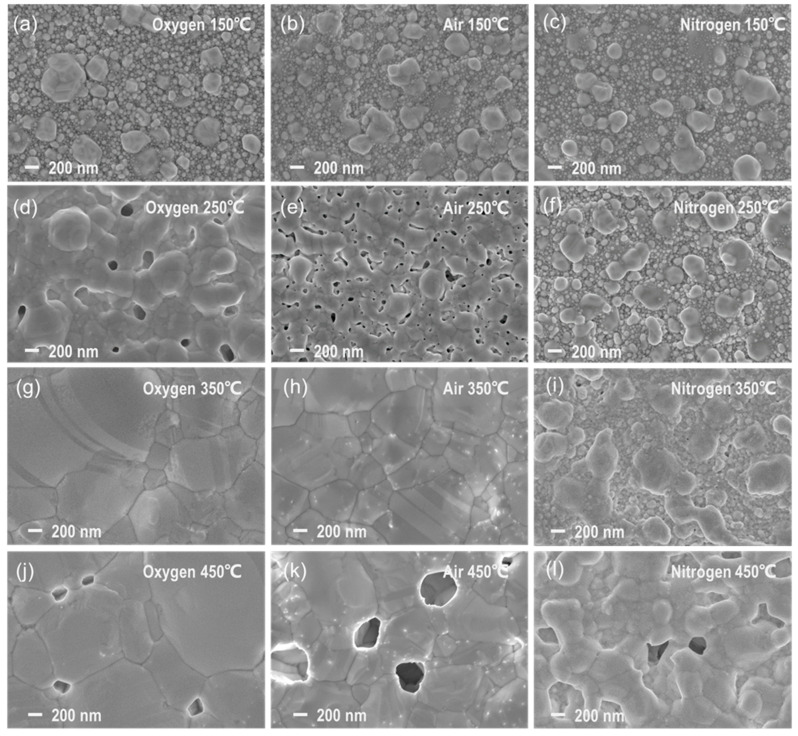
SEM images of the surface morphologies of silver films sintered under different atmospheres and temperatures. The columns from left to right correspond to oxygen, air, and nitrogen atmospheres, respectively. The rows from top to bottom correspond to 150, 250, 350, and 450 °C, respectively. (**a**–**c**) 150 °C; (**d**–**f**) 250 °C; (**g**–**i**) 350 °C; (**j**–**l**) 450 °C.

The original morphology of the silver NPs can be observed in Figure 4, where the SEM image of a sample after drying at 75 °C depicts silver NPs in the ink with a particle size of approximately 30–70 nm. Figure 5 shows the surface SEM images of the films sintered in oxygen, air, and nitrogen atmospheres. Compared with the original morphology of the silver NPs in the ink, the grain size of all the annealed films increased significantly, and the originally discrete NPs aggregated into larger grains. 

As can be observed in Figure 5, when the samples were sintered in pure oxygen, the morphology at 150 °C was composed of large grains above 100 nm in size inlaid with particles of the original size, as shown in Figure 5a. The morphology of the film sintered at 250 °C, shown in Figure 5d, demonstrated the coarsening of grains, whose size could reach more than 200 nm. Most of the grains were tightly connected to form an island-like microstructure, enabling the reduction in resistivity shown in Figure 3. The microstructure became denser with larger grains at 350 °C, as shown in Figure 5g, which corresponded to the lowest resistivity. When the sintering temperature rose to 450 °C, although the grain size was further enlarged, pores appeared on the surface, which might have caused the increase in resistivity. The morphological evolution of the samples sintered in air was quite similar to that in pure oxygen, and the main difference was that the grain sizes of the samples in air were smaller than those in pure oxygen, as shown in Figure 5b,e,h,k.

Comparing them with the morphologies of the samples sintered in the oxygen and air atmospheres, it could be noticed that the films sintered in nitrogen were quite different. As shown in Figure 5c,f,i,l, the grain coarsening and coalescence in nitrogen were much slower than those in the oxygen-containing atmospheres. Therefore, it could be supposed that the presence of oxygen promoted the aggregation process of NPs and thus enabled better film quality at lower temperatures.

**Figure 6:**
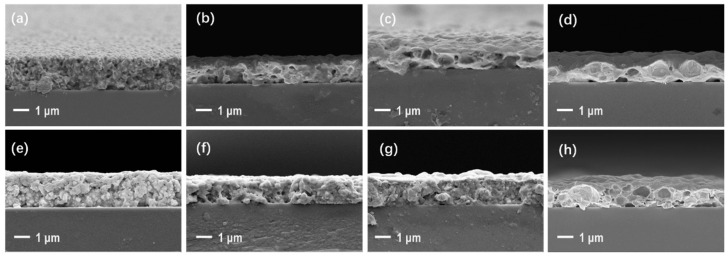
(**a**–**d**) SEM images of the cross-sections of silver films sintered in pure oxygen at 150, 250, 350, and 450 °C, respectively; (**e**–**h**) SEM images of the cross-sections of silver films sintered in pure nitrogen at 150, 250, 350, and 450 °C, respectively.

Figure 6 presents the cross-sectional SEM images of the sintered films. In pure oxygen, a cave-like microstructure was observed inside the film sintered at 150 °C, and the fine-graininess of the silver NPs was clear. The growth of larger grains was obvious at temperatures above 250 °C, and the interconnected structures could be observed, which are beneficial for reducing the film’s resistivity. 

Compared with those sintered in oxygen, the cross-sectional microstructures of the films sintered in pure nitrogen were much poorer. Especially at high temperatures, the films sintered in oxygen showed a compact microstructure, while those sintered in nitrogen displayed poor grain growth and many internal pores, to which the high resistivity of the films sintered in nitrogen shown in Figure 3 could be attributed.

**Figure 7:**
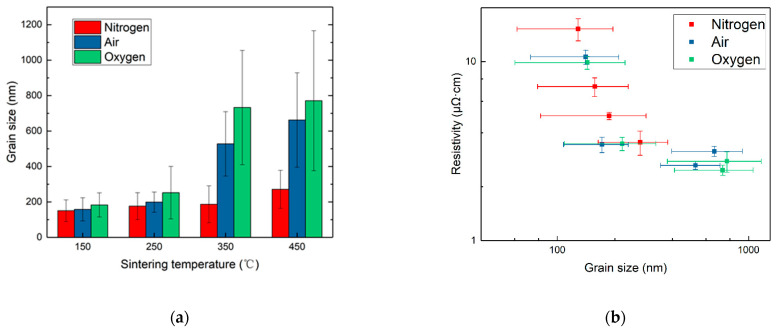
(**a**) The average grain sizes of films sintered under different atmospheres and temperatures; (**b**) the relationship between film resistivity and grain size considering all the samples in this study.

The average grain size was calculated by statistically analyzing the grain sizes in the SEM image, and it is illustrated in Figure 7a. The analysis was carried out using the SEM image analysis software Nano Measurer 1.2; the error bars represent standard deviation, and at least 100 dispersed grains were measured to ensure the accuracy. The grain size increased along with the sintering temperature for each atmosphere, consistent with the findings in Figure 5 and Figure 6. According to the report by Volkman et al. [45], a higher temperature could promote the coalescence process among NPs, resulting in the formation of larger grains during sintering. Jang et al. [46] found that the silver NP patterns sintered in air had larger grain sizes than those in nitrogen at the same temperature, and the larger grain sizes could contribute to a lower resistivity. The other factor promoting the coalescence of silver grains was the oxygen content in the sintering atmosphere. In pure nitrogen, the average grain size sintered at 350 °C was lower than 200 nm, while those in air and pure oxygen could be above 500 nm and 700 nm, respectively. 

The relationship between the mean grain size and the resistivity of the sintered films is illustrated in Figure 7b. The resistivity of the films sintered in all atmospheres gradually decreased along with the increase in grain size. When the grain size was below 200 nm, the resistivity curves basically overlapped, implying that the microstructural evolution under different atmospheres generally advanced along a similar route, although the progress in pure nitrogen required a higher temperature relative to those in oxygen and air. It could be speculated that the grain size was the dominant factor for the electrical resistivity when the organic dispersant was not decomposed.

When the grain size was further enlarged, the resistivity data under various atmospheres became scattered, indicating that the influence of factors other than grain size became significant. These factors might include the interface resistivity caused by residual organics and the newly formed pores in the film structure at high temperatures.

### 3.4. Organic Residue during Sintering

**Figure 8:**
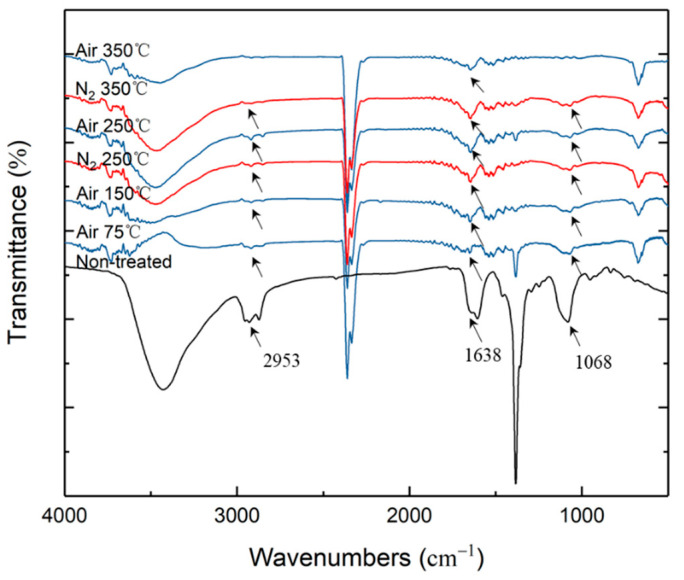
FTIR spectra of silver NP ink after sintering under air and pure nitrogen at various temperatures.

To reveal the changes in the organic residues in silver NP films sintered under different atmospheres and temperatures, FITR experiments were carried out. Figure 8 illustrates the FTIR spectrum of PVP and silver NP ink after sintering in air or nitrogen at a temperature of 75–350 °C. The peaks at 2953 cm^−1^ (C–H stretch) and 1638 cm^−1^ (C=O stretch) could be observed in the non-treated samples, which indicated the characteristic bands of PVP. In all the films sintered at temperatures below 250 °C, there was a peak at 1068 cm^−1^ (C–N stretch). 

Significant differences according to the atmosphere occurred when the temperature was raised to 350 °C. For the film sintered in air, the peaks of both the C–N stretch and the C–H stretch almost disappeared at 350 °C, indicating that PVP was decomposed. For the film sintered in nitrogen, these peaks remained. By using the normalized calculation relative to the sample dried at 75 °C, the absorbance values at the C–H and C–N stretch of the sample after sintering at 350 °C in air were only 2% and 8%, respectively. For the samples sintered in nitrogen, these values were 82% and 60%, respectively. 

## 4. Discussion

**Figure 9:**
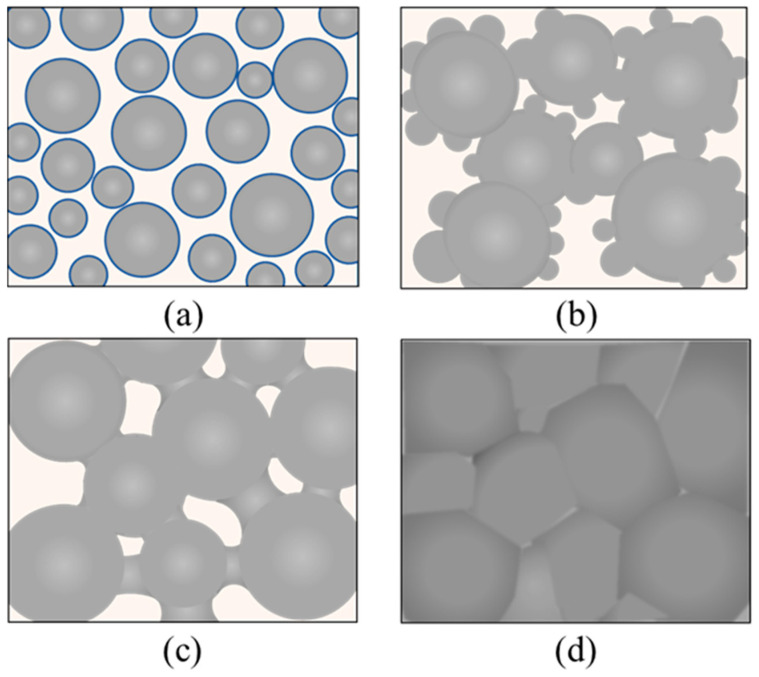
Schematic diagram of the sintering process of silver NPs in four stages: (**a**) initial stage; (**b**) Ostwald ripening stage; (**c**) particle coalescence stage; (**d**) densification stage.

Combining the observations in this study and the reports in the literature [47,48], the structural evolution of the grain morphology of silver NPs during sintering can be divided into four stages: the initial stage, the Ostwald ripening stage, the particle coalescence stage, and the densification stage, as depicted in Figure 9. In the initial stage, the silver NPs have a uniform grain size of tens of nm, and the organic dispersant PVP is capsulated around the NPs. When the temperature is raised, some grains can be enlarged due to the mechanism of Ostwald ripening, while their adjacent grains become smaller because of mass transfer and dissolution [49]. The driving force of Ostwald ripening is the system’s tendency to reduce its surface free energy, which is very high for a system composed of NPs [33]. The temperature is further raised to introduce the third stage of particle coalescence, which occurs when two or more silver NPs collide and merge to form a large particle [50]. The small grains can be completely absorbed into the large ones. The neighboring larger grains can form a “neck” and finally completely fuse together through the diffusion of silver atoms, resulting in a loose network structure for the entire film. Because the surface free energy of the silver grains is significantly decreased, the driving force of the Ostwald ripening is weaker, and the coalescence of the grains becomes the main mechanism of grain size enlargement at this stage. Continued heating can promote the further coarsening of the grains and the densification of the film surface, thus forming a compact network structure critical for reducing the electrical resistivity. However, it should be noted that overheating may occur if the temperature is too high, which produces pores that compromise the film’s conductivity, as illustrated by Figure 3 and Figure 5.

**Figure 10:**
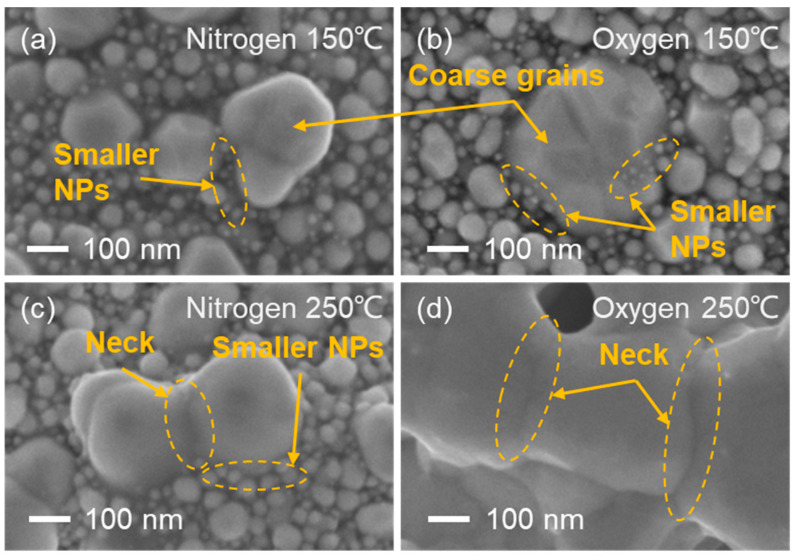
SEM images of microstructural difference between films sintered in different conditions. (**a**) nitrogen at 150 °C; (**b**) oxygen at 150 °C; (**c**) nitrogen at 250 °C; (**d**) oxygen at 250 °C.

A significant point that should be noted in this study is the differences caused by the various sintering atmospheres. As shown in Figure 5, Figure 6 and Figure 7, the differences in grain size and the microscopic morphology of the films sintered in pure nitrogen and oxygen-containing atmospheres indicated that oxygen content was beneficial for the formation of large grains at lower temperatures. The largest contrast could be observed in Figure 10. At 150 °C, the films sintered in nitrogen and oxygen were similar, and they were both in the second stage of Ostwald ripening, with the presences of coarse grains and smaller NPs. At 250 °C, the films sintered in oxygen were obviously in the third stage of particle coalescence, with wide necks, and all the smaller particles were consumed; however, the film sintered in nitrogen was still generally in the second stage, because many smaller NPs remained, although narrow necks had begun to appear. 

Therefore, the oxygen content in the sintering atmosphere could substantially enhance the grain enlargement, enabling the transition from the Ostwald ripening stage to particle coalescence at a lower temperature. In addition, it should be noted that such a stage transition in oxygen was accomplished at a temperature below 250 °C, when the decomposition of PVP had not yet begun according to the thermal analysis results shown in Figure 2. As reported by Yan et al. [51], the organic dispersant in silver NP ink had an important influence on the sintering process, because it was found through FTIR that the carboxyl oxygen atoms of PVP interacted with silver NPs on the surface. When the temperature was low, the particles were encapsulated by PVP, and the sintering mechanism at this time depended on surface diffusion; when the temperature was raised to decompose the dispersant, the sintering process turned to volumetric diffusion. However, the fact that the stage transition in oxygen was accomplished below 250 °C when the PVP was not decomposed implies that there was a difference in the mechanism of grain size enlargement within the scope of this study. In their study of silver thin film deposition [52], Presland et al. found that oxygen could increase the surface diffusion coefficient of silver atoms by hundreds of folds. They studied the formation of hillocks in the deposition of silver thin films and found that the diffusion flux of silver atoms during film growth was affected by oxygen partial pressure [53]. Jeong et al. confirmed that the mixing of oxygen helped to reduce the free energy of silver NPs during the coalescence stage, so that the evolution mechanism of the NPs changed, and the incomplete coalescence of silver NPs occurred earlier in the experimental group with excess oxygen, which promoted the rapid development of the silver film and significantly reduced the number and size of the pores in the silver film [54]. These phenomena are consistent with the evolution of silver NPs sintered below the decomposition temperature of PVP in this study. Silver in an oxygen-containing atmosphere has a stronger surface diffusion ability, and the surface diffusion and coalescence between particles are enhanced, so the films sintered in an atmosphere with a higher oxygen content have larger particles. Therefore, the effect of the oxygen content on improving the diffusion of silver atoms during sintering was significant even with the presence of the original PVP dispersant in the film. 

Moreover, the effect of the oxygen content on lowering the PVP decomposition temperature could contribute to the final sintering stage of film densification shown in Figure 10. As indicated by Figure 5, the temperature at which film densification could be finalized was approximately 350 °C for the oxygen-containing atmosphere and 450 °C for the pure nitrogen atmosphere. The temperatures of the initiation of PVP decomposition were about 280 °C in oxygen and 400 °C in nitrogen, respectively, as shown in Figure 2. As suggested in the related literature [33,34,35,36,37,38,39,40,41,42,43,44,45,46,47,48,49,50,51,52,53,54,55], the encapsulation of silver NPs by organic dispersants significantly affects the sintered morphology and consequently the properties of sintered silver thin films. Thus, it could be supposed that the breaching of the PVP dispersant capsulation of the silver NPs through enabling its decomposition is a prerequisite condition for the stage transition from particle coalescence to film densification, because the presence of PVP would limit the volumetric diffusion of silver NPs.

Besides the abovementioned effects of oxygen content, which could benefit our understanding of the sintering mechanisms of silver NPs, the findings in this study could also be instructive for engineering applications and equipment development for the ink-jet printing of silver thin films. First, as illustrated in Figure 3, the curves of electrical resistivity of the films sintered in air and pure oxygen were similar, and the micromorphological evolution of the films was also similar, as shown in Figure 5, indicating that the oxygen content in air was sufficient to ensure adequate grain growth from original silver NPs and PVP decomposition during sintering. The feasibility of using air as a sintering atmosphere instead of employing an oxygen-rich gas supply could help to simplify ink-jet printing equipment and lower its cost. Second, the optimal electrical conductivity could be achieved at approximately 350 °C in an oxygen-containing atmosphere, while such a temperature could damage the typical flexible substrates such as PET. Therefore, the application of heat for the practical sintering of as-printed silver circuits on a flexible substrate should be as brief as possible and concentrated on the silver NPs to avoid heat transfer from the silver film to the substrate. Based on the information discussed above, we developed an ink-jet printing system with a heating strategy that considers the absorbent properties of silver NPs and substrates and used it to fabricate high-performance samples, which will be reported in a forthcoming paper.

## 5. Conclusions

In this study, an investigation of the effect of the atmospheric oxygen content during the sintering of silver thin films derived from NP ink was carried out. The thermal properties of the silver NP ink and PVP dispersant were analyzed; the electrical conductivity of silver thin films sintered under different atmospheres and temperatures was compared; and the microstructure of the silver films and organic residue was analyzed. Combined with a discussion of the mechanisms of grain size and resistivity evolution, the following conclusions could be drawn:

(1) The thermal analysis of the NP ink and PVP showed that the decomposition temperature of PVP was above 400 °C in pure nitrogen, and the presence of oxygen in the sintering atmosphere lowered the onset temperature of PVP decomposition to 280 °C.

(2) The oxygen content significantly reduced the resistivity of the sintered thin films, and the optimal resistivity achieved in the film sintered at 350 °C in oxygen was 2.47 μΩ·cm, which is only 1.5-fold that of bulk silver. In addition, the resistivity–temperature curves of the films sintered in air and pure oxygen were quite similar.

(3) Compared with their counterparts in nitrogen, the silver NPs coalesced and coarsened much more rapidly when sintered in an oxygen-containing atmosphere, demonstrating that the oxygen content contributed to the enlargement of the silver grain size.

(4) The thin films sintered in an oxygen-containing atmosphere had less organic residue, which enhanced the silver diffusion to form a dense microstructure, thereby increasing the film’s conductivity.

## Data Availability

The data presented in this study are available from the corresponding authors upon a reasonable request.
